# Fetal skin as a pro-inflammatory organ: Evidence from a primate model of chorioamnionitis

**DOI:** 10.1371/journal.pone.0184938

**Published:** 2017-09-28

**Authors:** Suppawat Boonkasidecha, Paranthaman Senthamarai Kannan, Suhas G. Kallapur, Alan H. Jobe, Matthew W. Kemp

**Affiliations:** 1 Perinatal Institute, Cincinnati Children’s Hospital Medical Center, University of Cincinnati School of Medicine, Cincinnati, Ohio, United States of America; 2 Division of Neonatology, Department of Pediatrics, Queen Sirikit National Institute of Child Health, College of Medicine, Rangsit University, Bangkok, Thailand; 3 School of Women’s and Infants’ Health, The University of Western Australia, Perth, Australia; 4 Center for Perinatal and Neonatal Medicine, Tohoku University Hospital, Sendai, Miyagi, Japan; Xavier Bichat Medical School, INSERM-CNRS - Université Paris Diderot, FRANCE

## Abstract

**Background:**

Intrauterine infection is a primary cause of preterm birth and fetal injury. The pro-inflammatory role of the fetal skin in the setting of intrauterine infection remains poorly characterized. Whether or not inflammation of the fetal skin occurs in primates remains unstudied. Accordingly, we hypothesized that: **i)** the fetal primate skin would mount a pro-inflammatory response to preterm birth associated pro-inflammatory agents (lipopolysaccharides from *Escherichia coli*, live *Ureaplasma parvum*, interleukin-1β) and; **ii)** that inhibiting interleukin-1 signaling would decrease the skin inflammatory response.

**Methods:**

Rhesus macaques with singleton pregnancies received intraamniotic injections of either sterile saline (control) or one of three pro-inflammatory agonists: *E*. *coli* lipopolysaccharides, interluekin-1β or live *U*. *parvum* under ultrasound guidance. A fourth group of animals received both *E*. *coli* lipopolysaccharide and interleukin-1 signaling inhibitor interleukin-1 receptor antagonist (Anakinra) prior to delivery. Animals were surgically delivered at approximately 130 days’ gestational age.

**Results:**

Intraamniotic lipopolysaccharide caused an inflammatory skin response characterized by increases in interluekin-1β,-6 and -8 mRNA at 16 hours. There was a modest inflammatory response to *U*. *parvum*, but interleukin-1β alone caused no inflammatory response in the fetal skin. Intraamniotic Anakinra treatment of lipopolysaccharide-exposed animals significantly reduced skin inflammation.

**Conclusions:**

Intraamniotic lipopolysaccharide and *U*. *parvum* were associated with modest increases in the expression of inflammatory mediators in primate fetal skin. Although administration of Interleukin-1β alone did not elicit an inflammatory response, lipopolysaccharide-driven skin inflammation was decreased following intraamniotic Anakinra therapy. These findings provide support for the role of the fetal skin in the development of the fetal inflammatory response.

## Introduction

Preterm birth (delivery before 37 weeks’ gestation) is implicated in more than 1 million perinatal deaths worldwide each year [1]. Fetal and intrauterine inflammation cause preterm labor, and the presence of a systemic fetal inflammatory response at delivery is also associated with increased risk of adverse neonatal respiratory and central nervous system outcomes [2]. Identifying the gestational tissues responsible for generating intrauterine inflammation is thus an important step in advancing both our understanding of the aetiology of preterm birth and in the development of potential anti-inflammatory interventions.

A large literature describes the pro-inflammatory responses of the fetal lung and the chorioamnion to pro-inflammatory stimulation. However, the role of the fetal skin in driving intrauterine and fetal inflammation is less clear. The fetal skin is a large and highly vascularized immunologically competent organ [3, 4] that is continuously exposed to amniotic fluid *in utero*. Amniotic fluid can contain pathogenic microorganisms [5] and proinflammatory mediators associated with preterm labor and delivery including interleukins (IL)-1β, IL-6, IL-8 and tumour necrosis factor (TNF)-α [2, 6, 7]. Amniotic contents thus can interact with fetal skin by direct exposure, with the fetal gut epithelium by swallowing, and the fetal lung by fetal breathing and aspiration [8]. The skin has innate host defense systems to augment barrier function [9], but little is known about host defense in the skin of the preterm fetus, which in early gestation has minimal cornification and comparatively reduced barrier function [10].

Using sheep models of chorioamnionitis induced by the intraamniotic (IA) administration of *E*.*coli* lipopolysaccharide (LPS) or live *Ureaplasma parvum* (UP) we reported that the fetal skin responded with increased inflammatory cells and cytokine expression in the dermis [11–13]. With isolation of the oral and nasal epithelium and the fetal lungs and gut, an intra-amniotic exposure to LPS induced a systemic inflammatory response, likely mediated, at least in part, by inflammation in the fetal skin [14]. In contrast, exposure of only the fetal gut did not induce a systemic fetal inflammatory response [15].

There is very little information about how the fetal human or primate skin will respond to chorioamnionitis. We recently reported that intra-amniotic injection of LPS or IL-1β given to rhesus macaques with preterm pregnancies caused acute inflammation in the decidua, amniotic fluid, fetal lung and induced fetal inflammatory responses [16]. More chronic exposure to live *Ureaplasma parvum* caused minimal and inconsistent chorioamnionitis in the rhesus [17]. In the present report we asked if the fetal primate skin would mount a proinflammatory response to either LPS, live UP and recombinant IL-1β, three clinically relevant proinflammatory mediators associated with prematurity. We also sought to determine the importance of IL-1 signalling to a putative skin inflammatory response by the co-administration of LPS and the IL-1 receptor antagonist (IL-1ra) Anakinra.

## Materials and methods

### Animals

All animal procedures were approved by the Institutional Animal Care and Use committee at the University of California, Davis (18562) with use of approved standard operating procedures for Rhesus macaque (*Macaca Mulatta*). Time mated pregnant females were sedated with ketamine at approximately 125 to 130 days of gestation (term is 165 days) for ultrasound directed IA injections. Cesarean section deliveries followed sedation and pre-anesthesia for surgical field preparation and isoflurane anesthesia. Briefly, dams were sedated with an intramuscular injection of ketamine (10 mg/kg). The dams were then intubated, connected to an electrocardiogram monitor, and anaesthesia was maintained with inhalation isofluorane (1–2% fresh gas flow). Intravenous crystalloid solution (0.9% NaCl) was provided at a rate of 10 mL/kg/hour. Heart rate, respiratory rate, oxygen saturation, and depth of anaesthesia (jaw tone, corneal and palpebral reflexes, limb withdrawal reflex) were constantly monitored during the procedure. The fetus was removed in cull for recovery of gestational tissues and amniotic fluid, and euthanized with intravenous pentobarbital. The dam then received an intramuscular injection of atropine (0.04 mg/kg). Anaesthesia was withdrawn at the end of the procedure and dams were recovered with supplemental oxygen provided until airway control was regained. The dam’s appetite, hydration, stool quality, pain score, and incision site appearance was observed and recorded postoperatively for at least seven days. Intramuscular oxymorphone (0.15 mg/kg; administered between one and three times each day) was provided under veterinary direction based on clinical need.

The treatments were as follows:

1. **LPS:** LPS (1mg, E. coli O55:B5, Sigma Aldrich, Saint Louis, MO) in 1 mL of sterile saline solution was given by ultrasound-guided IA injection. After either 16 or 48 hours, fetuses were surgically delivered for fetal tissue collection;
2. **Ureaplasma:** Rhesus macaque dams were injected intra-amniotically with 10^7^ CFU *Ureaplasma parvum* (UP) serovar 1[17] under ultrasound gudiance. Fetuses were delivered surgically either 3 or 7 days later;
3. **IL-1β:** Rhesus macaque dams received 10 μg human recombinant IL-1β (Peprotech, Rockyhill, NJ) in 1 ml sterile saline or 1 ml sterile saline by ultrasound guided intra-amniotic injection [16] and were delivered either 1 or 3 days later; and
4. **LPS and IL-1ra:** LPS (1mg, E. coli O55:B5, Sigma Aldrich, Saint Louis, MO) in 1 mL of sterile saline solution was given by ultrasound-guided IA injection. 50mg IA and 100mg subcutaneous (SC) IL-1ra (Anakinra Sobi, Stockholm, Sweden) was given to pregnant monkeys at 1 and 3 hours, respectively before IA LPS administration. Fetuses were surgically delivered for fetal tissue collection after either 16 or 48 hours.

The variation in time to delivery between groups reflects the difference in the nature of the anticipated inflammatory response generated by different agonists, and to assess inflammatory responses at acute and also longer-term time points. Animals that were delivered 48 hours after IA LPS received a second IA + SC IL-1ra treatment, administered 24 hours after the first treatment. Matched saline controls were established for each of the four treatment groups. Samples of skin from the groin were fixed in 10% formalin for histological analysis, or snap frozen in liquid nitrogen for mRNA studies.

### mRNA quantitation

Total RNA was isolated from frozen skin samples by homogenization with TRIzol (Invitrogen, Carlsbad, CA) and column purification with RNeasy Universal Mini Kits (Qiagen, Valencia, CA) according to the manufacturer’s instructions. Reverse transcription was performed using Verso cDNA kit (Thermo Scientific, Waltham, MA). The relative expression of IL-1β, TNF-α, IL-6, IL-8, IL-10, IL-1α, IL-17α and monocyte chemoattractant protein 1 (MCP-1) was determined by rt-PCR using the cDNA template and rhesus macaque-specific Taqman probes and primers (Applied Biosystems, Foster City, CA). The mRNA expression for each gene was normalized to the mRNA for the 18s ribosomal protein as the endogenous control and a sample from the control group was used as a calibrator. Data are expressed as fold increase over the mean control value. Probe and primer set details are presented in Table 1.

**Table 1:** Summary of PCR sets.

TaqMan Probe	Assay ID (Applied Biosystems)	Entrez Gene ID
IL-1β	Rh02621711_m1	704701
IL-6	Rh02789322_m1	705819
IL-8	Rh02789781_m1	613028
TNF	Rh02789783_m1	715467
IL-10	Rh00961619_m1	694931
IL-1α	Rh02789770_m1	700193
IL-17A	Rh02621750_m1	708123
MCP-1	Rh02621753_m1	574138

### Immunohistochemistry

Immunohistochemistry was performed with 5 μm thick sections of formalin fixed tissues. Sections were de-paraffinized and rehydrated before microwave-assisted antigen retrieval in citric acid buffer at pH 6.0. Endogenous peroxidase activity was reduced with CH_3_OH/H_2_O_2_ treatment and the tissue was blocked with 4% normal goat serum in PBS. Sections were incubated overnight at 4°C with the primary antibody diluted in 4% serum in PBS. The following primary antibodies were used: anti-myeloperoxidase (MPO, Cell Marque 289A-75, cat. # 1404306A, dilution 1:200), anti-CD3 (Dako Cytomation, A0452 cat. # 20024875, dilution, 1:100), anti-CD 68 (Biosciences Pharmingen, M0814, cat. # 24648, dilution 1:200), anti-IL-8 (BD Bioscience, cat. # 550419, dilution, 1:100), anti-IL-6 (Lifespan Bioscience, Inc. LS-B7482 cat. # 37847, dilution, 1:400) and Anti-Pancytokeratin (Santa Cruz Biotechnology, SC-81714, cat. # B1015, dilution, 1:2500), with dilutions made in 4% normal goat serum. Sections were then washed and incubated with the appropriate species-specific secondary antibody diluted 1:200 in 4% serum for 2 hours at room temperature. After further washing, antigen:antibody complexes were visualized using a Vectastain ABC peroxidase kit (Vector Laboratories Inc., Burlingame, CA). Antigen detection was enhanced with nickel-diaminobenzidine, followed by incubation with Tris-1-cobalt. Slides were counterstained with Nuclear Fast Red for photomicroscopy. Blinded digital images of the fetal rhesus skin (5 non-overlapping, random fields per animal) were quantified for the total number of inflammatory cells, and immunostaining for IL-6, IL-8, neutrophil (MPO), monocyte/macrophage (CD68), and T-cells (CD3). Pancytokeratin staining intensity was also quantified by a blinded investigator, based on color threshold and expressed as percentage of intense, moderate and no staining area relative to total area.

### Cell counting

Sections stained for IL-6, IL-8, CD3 (T-cells), CD68 and MPO were counted for number of positive cells in ten randomly-selected fields per animal on photomicrographs (40x objective magnification) from the epidermal to dermal layers by an investigator blinded to treatment groups. A similar strategy was used to determine the percentage of cells positively stained for pancytokeratin (1:2,500), to determine both the number and the percentage cells with either intense, moderate or no staining.

### Statistical analyses

GraphPad Prism (GraphPad Software 5.Ink, La Jolla, CA) was used to graph and analyze data for statistical significance. Data are presented as means ± SEM (standard error of the mean). Data were tested for normality and variance; statistical differences between groups were analyzed using Mann–Whitney U tests. Values of p<0.05 were considered significant.

## Results

### Animals

There was no preterm labor or evident abnormalities in animals from either the four treatment groups or matched saline controls. Gestational ages at birth and birth weights were similar among the animals (Table 2). Mean fetal weights at tissue collection were not different between groups.

**Table 2:** *Treatment groups* Intra-amniotic injections of LPS, IL-1β, and *Ureaplasma parvum* (UP) or saline were performed in multiparous macaques of similar weight and age. Separate animals were used for controls for each treatment group. Mean fetal weights at tissue collection were not different between groups.

Treatment	N	Gestational age (days)	Birth Weight (gm)	Sex (M:F)
**LPS and IL-1ra treatment**				
Saline Control	13	132±0.6	337±8	6:7
LPS-16h	11	132±0.7	326±12	6:5
LPS-48h	8	132±0.8	320±10	1:7
LPS and IL-1ra-16h	6	132±1.5	354±14	3:3
LPS and IL-1ra-48h	3	128±0.8	315±9	3:0
**IL-1β treatment**				
Saline Control	5	131±1.1	310±9	0:5
IL-1β- 1d	5	130±0.7	341±13	3:2
IL-1β- 3d	4	129±0.3	346±38	1:4
**UP treatment**				
Saline Control	5	132±0.9	343±4	3:2
UP-3d	5	130±1.1	348±19	2:3
UP-7d	5	130±0.5	341±8	2:3

### IA LPS, UP but not IL-1β increase cytokine expression in the fetal rhesus skin

**Fig 1:**
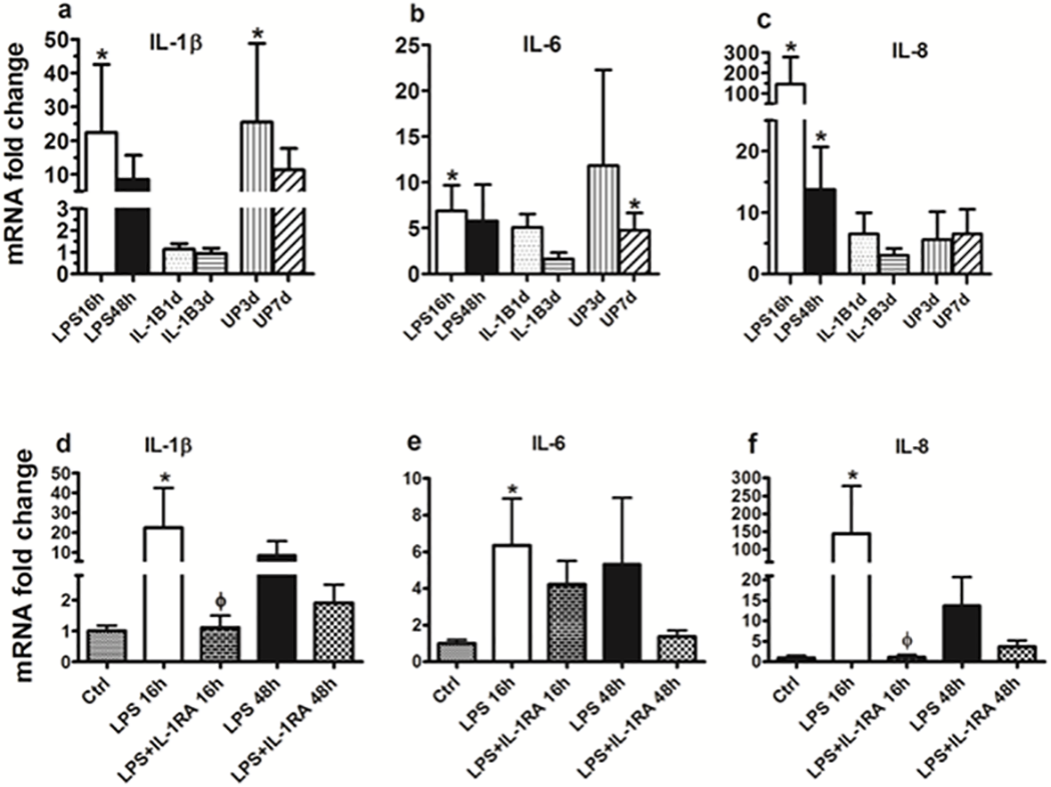
Cytokine PCR Analysis. Quantitative PCR analysis of cytokines in fetal rhesus macaque skin following intra-amniotic exposure with LPS, IL-1β or UP and for different times of exposure (Panel a, b and c). Cytokine expression for LPS and LPS plus IL-1ra exposure groups (Panel d, e and f). Graphs represent mean mRNA fold change ± SD. *indicates a significant increase in transcript expression relative to untreated control group normalized to a value for 1.0. ɸ indicates a significant decrease in transcript expression relative to LPS at 16 hours.

IL-1β, IL-6 and IL-8 mRNA significantly increased 16 hours after IA LPS (p<0.05). Only IL-8 mRNA remained significantly elevated 48h after LPS exposure (Fig 1, panels a-c). The cytokines TNF-α, IL-1α, and MCP-1 did not change with LPS exposure, and IL-10 was undetectable in association with LPS exposure. In the UP treatment group, IL-1β mRNA was significantly increased by 25-fold at 3 days and IL-6 mRNA increased by 5-fold at 7 days after IA UP (p<0.05, Fig 1, panels a and b). In contrast to LPS exposure, TNF-α increased significantly 15 fold at 3 days and 9 fold at 7 days, but IL-10, IL-1α, and MCP-1 did not change with UP exposure (Table 3). IL-1β treatment did not significantly increase the mRNA expression of any of the cytokines or chemokines measured at either time point.

**Table 3:** Summary of cytokine / chemokine mRNA expression. Data are mean ± SD; *p = 0.05. Cells without data represent transcript levels below limit of detection.

	TNF	IL-10	IL-1α	MCP-1
**Control**	1.0±0.6	1.0±1.0	1.0 ±0.8	1.0±0.9
**LPS 16h**	1.3±0.8	-	2.5±3.9	1.4±0.8
**LPS+IL-1RA 16h**	1.2±0.6	-	1.8±1.4	1.8±0.6
**LPS 48h**	3.1±5.7	-	1.3±0.8	1.0±0.8
**LPS+IL-1RA 48h**	3.3±3.4	-	3.7±3.2	1.2±0.6
**IL-1B 1d**	1.7±1.4	2.3±2.6	1.7±1.7	0.7±0.3
**IL-1B 3d**	1.1±0.5	0.6±0.3	0.7±0.3	0.3±0.1
**UP 3d**	15.0±29.6*	-	1.4±1.0	2.4±2.6
**UP 7d**	9.0±8.9*	-	1.2±0.5	2.7±2.1

### IL-1ra decreased IL-1β and IL-8 mRNA expression in LPS exposed fetal rhesus skin

In the LPS + IL-1ra treatment group, there were statistically significant decreases in IL-1β and IL-8 mRNA expression at 16 hours (p<0.05, Fig 1, panels d and f) relative to LPS-treatment only.

### IA LPS and the impact of IA IL-1ra on inflammatory mediators

**Fig 2:**
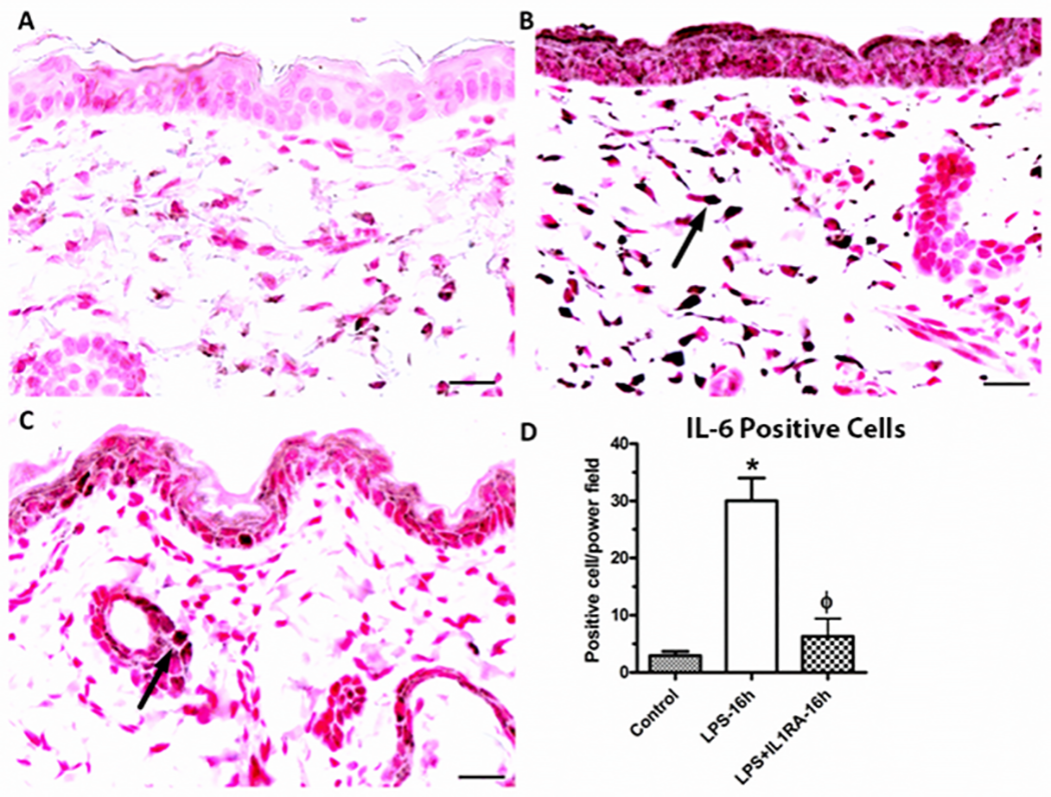
IL-6 expression. Representative photomicrographs for IL-6 (1:400) immunohistochemical staining of fetal skin. (A) control; (B) LPS at 16 h; (C) LPS and IL-1ra at 16 h exposure. (D) The number of positive cells (arrow), that were counted between groups. (magnification bar represents 20 μm). *indicates a significant increase in the number of positive cells relative to the untreated control. ɸ indicates a significant decrease in number of positive cells relative to LPS.

**Fig 3:**
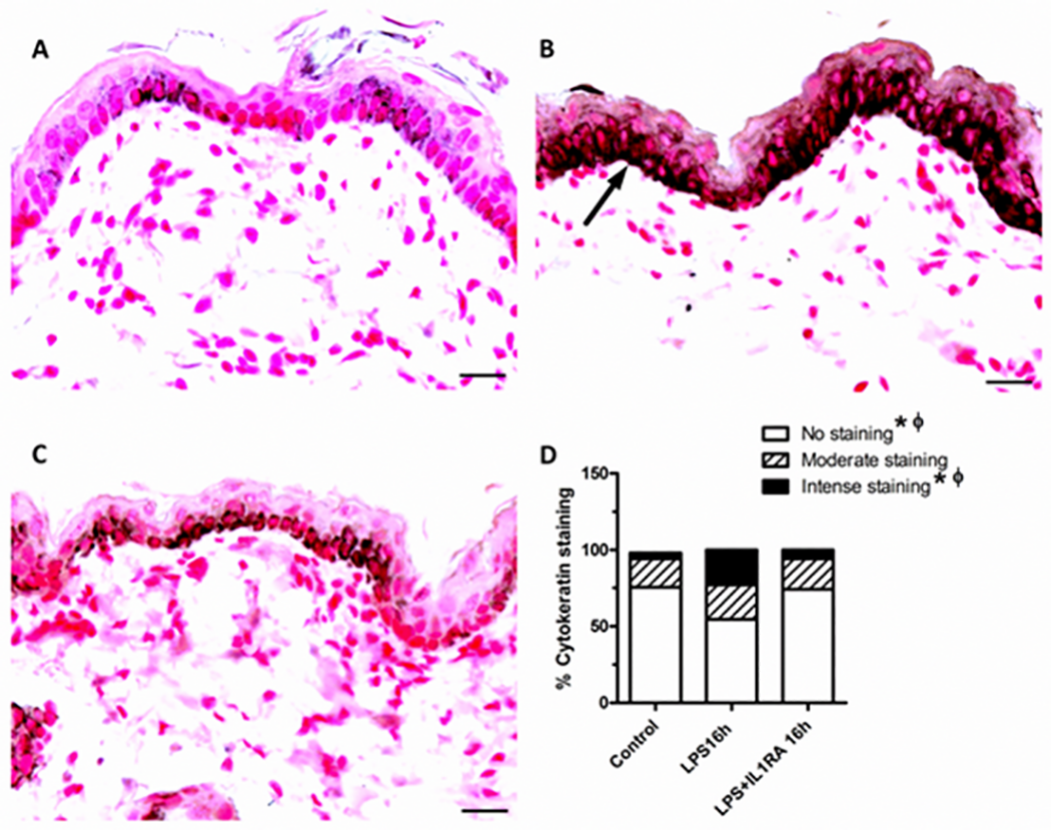
Pancytokeratin expression. Representative photomicrographs for pancytokeratin (1:2500) immunohistochemical staining of fetal skin. (A) control; (B) LPS at 16 h; (C) LPS and IL-1ra at 16 h exposure. (D) Graph shows the comparison between groups in the percent of positive cells of different intensity stain; intense (arrow), moderate and no stain (magnification bar represents 20 μm). *indicates a significant difference in the percent of positive cell between LPS at 16 hours and untreated control group. ɸ indicates a significant difference in the percent of positive cell between LPS group and LPS plus IL-1ra group at 16 hours.

We performed immunohistochemistry (IHC) for IL-6, IL-8, CD3, CD68, MPO and pancytokeratin. The number of cells positively stained for IL-6 and pancytokeratin increased significantly 16 hours after IA LPS (p<0.05, Figs 2 and 3). We did not identify changes in the other markers assessed. The administration of IL-1ra significantly decreased the number of IL-6 positive cells (p<0.05, Fig 2). There was a significant increase in the percentage of intensely positive pancytokeratin stained cells for the LPS group at 16 hours relative to the untreated control group. At 16 hours, there was significant decrease in the percent of intense positive cell after IA IL-1ra administration (p<0.05, Fig 3). There was no difference in moderate staining of pancytokeratin IHC between groups. We did not observe infiltrating neutrophils (MPO), leukocytes (CD3+) or infiltrating macrophages (CD68+) in fetal skin after IA LPS treatment.

## Discussion

We demonstrate that the fetal rhesus skin is capable of generating an inflammatory response *in utero*, in response to direct stimulation by both LPS and UP. In LPS-exposed animals, we show that this response involves increased expression of mRNA transcripts for preterm birth-associated proinflammatory cytokines IL-1β, IL-6, and IL-8, and increased staining for IL-6 and pancytokeratin in the skin. This observation is consistent with previous reports of increased expression of cytokeratin following stimulation of keratinocytes with pro-inflammatory cytokines [18]. IA UP-treatment resulted in increased IL-1β mRNA expression at 3d post-treatment and increased IL-6 expression at 7d post-treatment. Although these studies were not designed to accurately determine the ontogeny of skin responses to inflammatory agonist, it is apparent that the skin is able to rapidly mount an inflammatory response to microbial agonist and, that in the case of UP, this response persists for an extended period. In our experimental system it is not possible to isolate the impact of systemic vs direct agonist inflammatory stimulation on the fetal skin.

IA administration of IL-1β alone was not associated with pro-inflammatory changes in the fetal skin. However, the co-administration of IL-1ra with LPS was associated with significant reductions in IL-1β and IL-8 transcript expression, in the number of IL-6 positive cells in the dermis, and in the intensity pancytokeratin staining relative to LPS-treatment only. Taken together, these results demonstrate that both LPS and UP can induce fetal skin inflammation, and, given that LPS-driven inflammatory responses are partially blocked with IA IL-1ra therapy, that IL-1 signaling likely plays an important role in skin inflammation *in utero*.

Infection of amniotic fluid results in the production of proinflammatory cytokines and chemokines [19, 20]. The fetal skin, which has a large surface area, is entirely exposed to the amniotic fluid containing any invading microorganisms and proinflammatory agonists. Important questions that remain unanswered are the relative importance of maternal or fetal inflammation, and which tissues (chorioamnion, placenta, fetal lung or fetal skin) are primarily responsible for initiating the fetal inflammatory responses.

We suggest that the developing fetal skin may contribute to the acute fetal inflammatory response (FIRS), which is associated with preterm birth and adverse neonatal outcomes. Even a limited inflammatory fetal skin response could have systemic effects on the fetus due to a large surface area and the high degree of vascularization. We previously showed in fetal sheep that chorioamnionitis caused by the intra-amniotic injection of LPS resulted in inflammation, particularly of the fetal lung, gut, skin, and chorioamnion [13, 21, 22]. IL-1 was central to this inflammation as blockade of IL-1 signaling in the amniotic compartment with the same recombinant IL-1 receptor antagonist (IL-1ra) largely inhibited the fetal lung and systemic inflammation caused by intra-amniotic *E*. *coli* LPS [20]. The skin inflammatory response to UP were modest but the evaluation was 3 and 7 days after IA injection of organisms. We anticipate that there would be a longer interval from IA injection to an inflammation for UP than LPS.

The proinflammatory cytokines IL-1β and TNF-α are considered primary mediators of septic shock and are also associated with infection-induced preterm delivery [16]. The robust expression of IL-1 β by the primate fetal skin in response to IA LPS stimulation may be of significance, given the association of these cytokines with infection-associated preterm labor. Increases in IL-6 expression are frequently identified in association with preterm birth. IL-6 is a marker of systemic fetal inflammatory response syndrome (cord blood IL-6 >11 pg/mL) [2, 6, 19]. In the present study, IL-6 transcript expression in the fetal skin was increased significantly only after 16 hours of LPS stimulation, but remained elevated 7d after UP exposure. IL-8 is a potent chemotactant and neutrophil activating factor which is part of the response elicited in the host against microbial invasion.

IL-8 also can recruit neutrophils to the fetal membranes and the placenta during the development of an intrauterine infection. There was no significant change in IL-8 protein expression in response to UP administration at either 3d or 7d. Although IL-8 transcript expression in the fetal skin was increased significantly both 16 and 48 hours post-LPS stimulation, we did not observe infiltrating neutrophils (MPO), leukocytes (CD3+) or infiltrating macrophages (CD68+) in fetal rhesus skin as we anticipated.

A possible explanation for these findings may be the structural immaturity of the fetal skin which may allow immunocytes to move from the skin to the amniotic fluid. Previous studies have suggested that fetal immunocytes (specifically, neutrophils, lymphocytes and monocytes) are recruited into the amniotic fluid [22]. After IA LPS administration, immunocytes isolated from the amniotic fluid expressed proinflammatory cytokine mRNA for IL-8 at 72 hours and 7 days while no cytokine mRNA can be detected in the chorioamnion, lung, or spleen after 72 hours [22]. The permeable nature of the developing fetal rhesus skin at early gestation may be permissive for the transepidermal passage of cytokines and chemokines from the fetus into the amniotic fluid [22].

In the present study, an injection of IL-1β sufficient to induce chorioamnionitis and fetal inflammatory response [16] did not cause skin inflammation in the non-human primate. In a related unpublished observation, IA IL-1β also did not cause skin inflammation in sheep (M. Kemp, Personal Communication). This observation is of interest, given the importance of IL-1 signaling to skin inflammation, and the robust response seen in the macaque fetal lung and chorioamnion in response to IL-1β stimulation. IL-1 is well established as a critical mediator of skin inflammation, with both IL-1α and IL-1β acting via IL-1 receptor 1. Enk and colleagues, for example, have proposed that IL-1β produced by dendritic cells may be critical to the induction of skin immune responses[23]. Dysregulation of the IL-1α: IL-1Ra ratio has been implicated in a range of inflammatory skin diseases including psoriasis and contact dermatitis [24].

Biologically active 31 kDa IL-1α is the predominant form expressed by epithelial cells, including keratinocytes. In contrast, biologically active IL-1β is generated by cleavage of the 31 kDa inactive form by stimulated Langerhans cells, monocytes and macrophages. Although keratinocytes also generate the biologically inactive 31kDa IL-1β precursor, they largely lack the enzyme necessary to complete significant post-translational generation of the mature 17 kDa protein [25].

It is tempting to speculate as to why 10 ug of IA IL-1β failed to elicit skin inflammation in the macaque fetus, despite being associated with pulmonary inflammation and chorioamnionitis. Firstly, it is possible that there was insufficient IL1R available in the fetal skin to drive a detectable inflammatory response to IL-1β stimulation. Using newborn human foreskin samples, Groves and colleagues demonstrated that in contrast to adult human skin (which demonstrates robust basal and apical epidermal IL-1R staining [26]) untreated tissues expressed very few IL-1R (either type 1 or 2), although significant and rapid increases in the expression of the type-II (inhibitory) IL-1R could be induced by tissue stimulation with interferon-ɣ, or by overnight incubation at 37°C [27]. In contrast, Toll-like receptors (including Toll-like receptor 4, which is strongly agonized by LPS) are expressed in skin from an early gestational age, likely facilitating the LPS-driven response identified in the present report[28]. However, against this hypothesis is our observation that IL-1Ra was able to antagonize LPS-driven inflammation, which itself requires the binding of IL-1Ra to type I IL-1R.

Secondly, it is possible that the dose of IL-1β administered was insufficient to overcome inhibition of IL-1R1 signaling by endogenously expressed IL1Ra, although given the high ration of IL-1Ra: IL1 required to inhibit productive signaling (ranging between 10:1 and 100:1 [24]), this explanation also seems unlikely. Thirdly, it is possible that the magnitude of inflammatory response generated by IL-1β stimulation were too small to be identified statistically with the group sizes used in our study. Lastly, unlike in the fetal lung, it is possible that IL-1β-driven responses in the skin are acute, modest, and resolve within 24h.

There are a number of clinical correlations of this study. In chorioamnionitis-induced fetal inflammatory response, the fetal skin may contribute to the inflammation in the amniotic fluid and systemically. Furthermore, skin inflammation could further compromise the already poor barrier function of the fetus or decrease the permeability by the increased cornification. Infants exposed to chorioamnionitis could have an increased predisposition to nosocomial infection from alterations in skin barrier function. IA IL-1ra therapy can block the inflammatory responses in primate fetal skin and FIRS from LPS, the receptor blockers may both block preterm delivery and fetal injury.

In conclusion, the primate fetal skin can mount an inflammatory response to *in utero* inflammation, although this response is modest and appears to lack an immunocyte response, at least acutely. However, given the size, degree of vascularization, and direct exposure to the amniotic fluid, the primate fetal skin may be an important source of the both fetal inflammation and pro-inflammatory mediators that could signal the chorioamnion, cervix, and uterus to ultimately induce preterm labor.

## Abbreviations

IA: Intraamniotic
LPS: lipopolysaccharides
UP: *Ureaplasma* *parvum*
IL: Interleukin
TNF: Tumour necrosis factor
CFU: Colony forming unit
CCL2: Chemokine ligand 2
ELISA: Enzyme-linked immunosorbent assay
MCP-1: Monocyte Chemoattractant Protein-1
MPO: Myeloperoxidase
PBS: Phosphate buffer saline
